# Evaluation of GABA Receptors of Ventral Tegmental Area in Cardiovascular Responses in Rat

**Published:** 2014-07

**Authors:** Minoo Rasoulpanah, Fathmeh Kharazmi, Masoumeh Hatam

**Affiliations:** 1 Department of Physiology, School of Medicine, Shiraz University of Medical Sciences, Shiraz, Iran;; 2 Department of Physiology, School of Medicine, Hormozgan University of Medical Sciences, Bandar Abbas, Iran

**Keywords:** Ventral tegmental area, GABA, Blood pressure, Heart rate

## Abstract

**Background: **The ventral tegmental area (VTA) is well known for its role in cardiovascular control. It is demonstrated that about 20-30% of the VTA neurons are GABAergic though their role in cardiovascular control is not yet understood. This study is carried out to find the effects of GABA A and GABA B receptors on cardiovascular response of the VTA.

**Methods:** Experiments were performed on urethane anesthetized male Wistar rats. Drugs were microinjected unilaterally into the VTA. The average changes in mean arterial pressure (MAP) and heart rate (HR) were compared between the case and the control groups using *t* test and with the pre-injection values using paired *t *test.

**Results: **Microinjection of muscimol, a GABAA agonist (500, 1500 and 2500 pmol/100nl) into the VTA had no significant effect on MAP and HR compared with the saline group and pre-injection values. Injection of bicuculline methiodide (BMI, 100 and 200 pmol/100 nl), a GABAA antagonist, caused a significant increase in the MAP (11.1±1.95mmHg, P<0.5) and a decrease in HR (-32.07±10.2, P<0.01). Microinjection of baclofen a GABA_B_ receptor agonist (500 or 1000 pmole/100 nl) and phaclofen a GABA_B_ receptor antagonist (500 or 1000 pmole/100 nl) had no significant effects on MAP and HR.

**Conclusion: **For the first time it was demonstrated that GABA system of the VTA inhibits the cardiovascular system through the activation of GABAA but not the GABAB receptors.

## Introduction

The ventral tegmental area (VTA) is a part of mesolimbic dopamine system located in the medial portion of the ventral mesencephalon. The VTA can be divided into rostral and caudal sub-regions. The VTA is one of the dopaminergic (DA) origins in the central nervous system^1^^,^^2^ and is well known for its role in cardiovascular regulation.^3^^-^^5^ For instance, chemical stimulation of the VTA with L-glutamate decreases both arterial pressure (BP) and heart rate (HR) mediated by an inhibition of sympathetic but not the parasympathetic fibers.^6^ Microinjection of substance P analogue, Dime-c7 into the VTA evokes dose-dependent increase in BP and HR. Intravenous pretreatment with dopamine D1 and D2 receptors antagonists markedly inhibited Dime-c7 effects as well as intravenous administration of vasopressin receptor antagonist. This suggests that an increase in vasopressin release is contributing to the rise in BP in response to Dime-c7 injection.^7^^,^^8^ The pressor response of VTA to stimulation by substance P analogue is prevented by either bilateral electrolytic or chemical lesions of supraoptic nucleus of hypothalamus.^9^

Miroinjection of tachykinin receptor agonists of NK1, NK2 and NK3 into the VTA are shown to increase BP and HR. These responses are inhibited by intravenous treatment with antagonist for D1 dopamine receptors and β1 adenoreceptor.^10^ GABA injection into the VTA attenuated pressor response to administration of angiotensin II into lateral cerebral ventricle.^11^ Injections of neurotensin into the VTA have no effect on mean arterial pressure and heart rate but significantly potentiated the pressor response to intravenous administration of vasopressin. This effect is blocked by intravenous pretreatment with raclopride.^8^

The DA neurons comprise 70-80% of all neurons in the VTA^12^ and the GABAergic neurons comprise about 20-30% of the VTA neurons.^12^^-^^15^ The VTA receives GABAergic projection from ventral pallidum and nucleus accumbens^16^ and ascend to nucleus accumbens^17^ and prefrontal cortex.^18^ The DA neurons receive GABAergic input from local GABAergic interneurons too.^19^ Electrophysiological studies have shown that GABAB receptors in the VTA hyperpolarizes dopamine neurons.^2^ Other evidence suggests that dopamine neurons are under tonic inhibition of the GABAergic afferents.^20^ On the other hand, receptors of some neurotransmitters and neuropeptides are expressed on the local GABAergic neurons. For example, acethylcholine through its muscarinic receptor increases the frequency of spontaneous inhibitory postsynaptic current (IPSC's) in GABAergic neurons.^19^ Activation of muscarinic receptors presynaptically inhibits the amplitude of GABAergic synaptic currents.^19^^,^^21^

Considering the above, this study was performed to find the role of GABAergic system of VTA in cardiovascular regulation and to assess the role of each GABA receptor subtype by microinjection of selective agonists and antagonists into the VTA in urethane anesthetized male rats. 

## Materials and Methods

*Subjects and Surgery*

The experiments were performed on 62 male Wistar rats (200-300 g) in accordance and approval by the Animal Use and Care Committee of Shiraz University of Medical Sciences. The animals were randomly divided into six groups and were anesthetized with urethane (1.4 g/kg, ip) and supplementary doses (0.7 g/kg) were given as required. The trachea was cannulated to ease ventilation and their body temperature was maintained at 37±1° C by an animal temperature controller (Narco-Bio. System, USA). The femoral artery was cannulated with polyethylene catheter (PE-50) filled with heparinized saline and the catheter was connected to a pressure transducer (Harvard). The arterial pressure and heart rate were continuously recorded by both a Harvard polygraph and a computer program (written by Dr A. Nasimi).  In some experiments, femoral artery was connected to a ML T844 pressure transducer coupled to a pre-amplifier (FE221 bridge amplifier) connected to power lab 4/35 data acquisition system (model PL3504AD-instruments). The animals were placed in prone position in a stereotaxic frame (Stoelting, USA) and two small holes were drilled through the parietal bone over the VTA.

*Drugs Microinjection*

Unilateral microinjection of drugs was performed by a micropipette with 35-45 µm internal diameter. Micropipette tips were positioned in the VTA according to a stereotaxic atlas of the rat brain.^22^ The stereotaxic coordinates of the VTA were explored from 4.6 to 5.2 mm caudal to bregma, midline to 0.7-1.3 mm lateral and 8.2-8.4 to ventral from the bregma. Pressure injection was performed by a pressurized air pulse applicator and the volume of injection was measured by direct observation of the fluid meniscus in the micropipette by using a microscope fitted with an ocular micrometer (made in U.W.O, Canada). The injection volume of all the drugs was 100nl.

All drugs except phaclofen were dissolved in saline. Phaclofen was dissolved in 4% of HCL 0.1 N and 96% of saline, and the PH was raised to seven by adding NAOH. The injection sites were 100 µm apart and 1-3 injections were made in each animal on both sides.

*Experimental Groups*

The experiments for studying the GABAergic system of VTA consisted of 6 groups as follow:

-Microinjection of saline or vehicle of phaclofen (n=9 rats, injections=12) as the control groups. All procedures were exactly the same as the experimental groups but instead of drugs, the vehicle was injected into the VTA. 

-Microinjection of three doses of a GABA_A_ agonist, muscimole,(0.5 nmol/100nl; 1.5 nmol/100nl and 2.5 nmol/100 nl; n=10 rats, injections=28; Sigma)

-Microinjection of two doses of a GABA_A_ antagonist, bicuculline methiodide (BMI, 100, 200 pmol/100 nl; n=24 rats, injections=47; Sigma)

-Microinjection of two doses of a GABA_B_ agonist baclofen (500, 1000, pmol/100 nl, n=6 rats, injections=13; Sigma).

-Microinjection of two doses of a GABA_B_ antagonist, phaclofen (500,1000, pmol/100nl; n=17 rats, injections=30, Sigma).

*Histology*

At the end of each experiment, the animal was sacrificed by a high dose of the anesthetic and then perfused transcardially with 100 ml of 0.9% saline followed by 100 ml of 10% formalin. The brain was removed and stored in 10% formalin for at least 24-h. Frozen serial transverse sections (50 micron) of the brain were cut and stained with cresyl violet 1%. The injection sites were determined according to a rat brain atlas^22^ under microscope light.

*Data Analysis*

The results are expressed as mean±standard error of mean (SE). Initially Kolmogorov-Smirnov normality test was performed on data and all were found to be normal. 

The mean of the maximum changes of the heart rate and the mean arterial pressure were compared with those of the control group using independent *t*-test and with the pre-injection values using paired *t*-test. A P<0.05 was taken to indicate statistical significance.

## Results

*Cardiovascular Responses to Vehicle Microinjected into the VTA*

Microinjection of vehicle (saline or phaclofen solvent) did not affect the pressure or the heart rate (saline ∆MAP=0.16±0.58 mmHg, ∆HR=-0.58±1 bpm; phaclofen solvent ∆MAP=0.35±0.17 mmHg, ∆HR=-0.45±0.22 bpm).

*Cardiovascular Responses to Muscimol Microinjected into the VTA*

**Figure 1:**
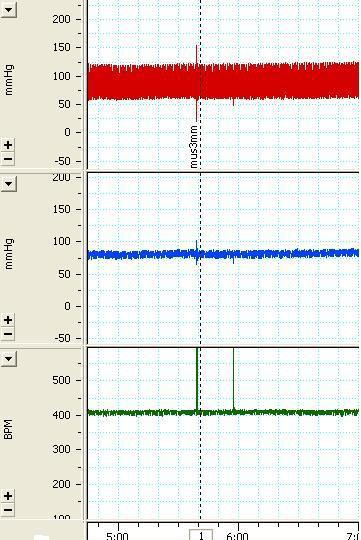
The tracing of the arterial pressure (mmHg) and heart rate (beat per minute, bpm) depicting the effects of unilateral microinjection of muscimol 2.5 nmol/100 nl the arrow indicates the injection time.

**Figure 2:**
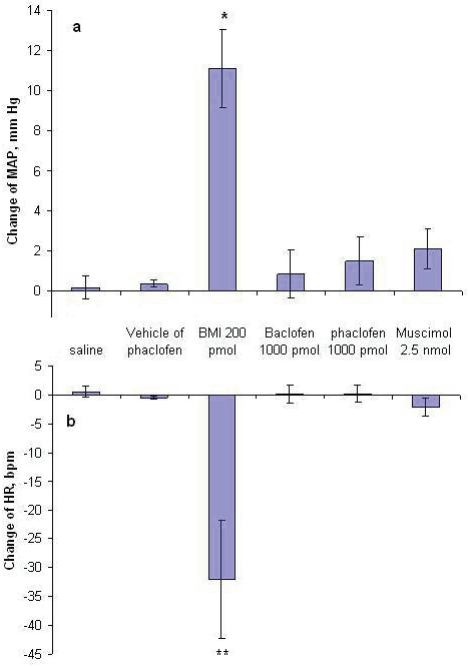
The effects of bicuculline 200 nmol/100 nl, muscimol 2.5 nmol/100 nl and baclofen 1000 pmol/100 nl and phaclofen 1000 pmol/100 nl on MAP (a) and HR (b) compared to the control values. *P<0.5; **P<0.01* t* test

To study the effect of GABA on the cardiovascular responses, different doses of muscimol, a GABAA agonist were injected into the VTA (basal MAP=96.4±3.8 and basal HR=404.5±5.8). Tests carried out included 500 pmol/100 nl (∆MAP=-2.5±4.4 mmHg, ∆HR=-0.2±1.9 bpm, n=3 rats, 9 injections) 1.5 nmol/100nl (∆MAP=3.1±1.5 mmHg, ∆HR=6.0±3.1 bpm, n=4 rats, 11 injections) and 2.5 nmol/100 nl (∆MAP=2.1±1.5mmHg, ∆HR=-2.1±1.6 bpm, n=3 rats, 8 injections). None of the above produced a significant change compared with the saline group and pre-injection values. A sample of the arterial pressure and HR tracings before and after the injection 2.5 nmol/100nl muscimol are shown in figure 1 and the maximum MAP and HR changes are shown in figure 2.

*Cardiovascular Responses to BMI Microinjected into the VTA*

Different doses of bicuculline methiodide (BMI), a GABA_A_ antagonist, were injected into the VTA of anesthetized rats (basal MAP=93.8±4.8 and basal HR=369.6±7.9). The injections were; 100pmol/100nl (∆MAP=11.1±1.95mmHg, ∆HR=-32.07±10.2 bpm, n=14 rats, 28 injections) and 200 pmol/100 nl (15.4±5.8 mm Hg, ∆HR =-113.6±32.6 beats/min n=10 rats, 19 injections).

**Figure 3:**
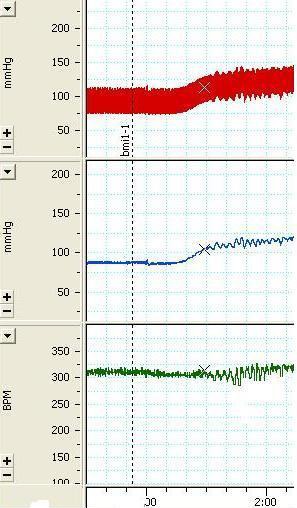
The tracing of the arterial pressure (mmHg) and heart rate (beat per minute, bpm) depicting the effects of unilateral microinjection of bicuculline 200 nmol/100 nl the arrow indicates the injection time.

Figure 3 shows a sample of the arterial pressure and HR tracings before and after the injection of 200 pmol/100 nl BMI. As shown, BMI increased MAP and decreased HR in the majority (70%) of the cases. In this group, the range of the MAP change was from 5 to 57 mm Hg and the range of the bradycardia was from -10 to -290 beats/ min. The variations reached a peak at 3 to 10 minutes and returned to baseline at most in 30 minutes. In some experiments, high doses produced higher hypertension and bradycardia which returned to baseline after around 1h. Compared with the baseline values, BMI caused a peak significant increase in the MAP (paired *t *test, P<0. 05) and significant decrease in the HR (paired *t-*test, P<0.001). Also the maximum MAP and HR changes were significantly (independent *t *test, P<0.01) different from that of the saline group (figure 2).

*Cardiovascular Responses to Baclofen Microinjected into the VTA*

**Figure 4:**
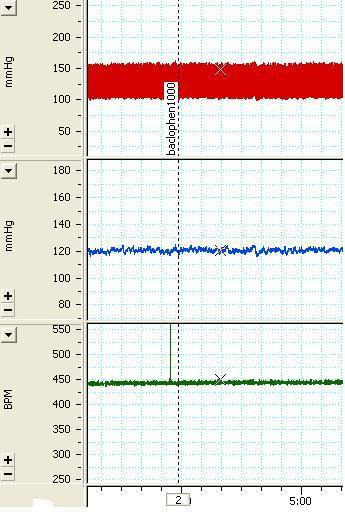
The tracing of the arterial pressure (mmHg) and heart rate (beat per minute, bpm) depicting the effects of unilateral microinjection of baclofen 1000 pmol/100 nl the arrow indicates the injection time.

Three doses of baclofen, a GABA_B_ receptor agonist, were injected into the VTA (basal MAP=96.1±4.5 and basal HR=372.7.5±13.5). Tests carried out included; 500 pmol/100 nl (∆MAP=-1.14±0.59 mmHg, ∆HR=-0.85±1.8 bpm, n=3 rats, 9 injections), 1000pmol/100 nl (∆MAP=0.85±1.2 mmHg, ∆HR=0.14±1.62 bpm, n=3 rats, 6 injections) and 2.5 nmol/100 nl (∆MAP=2.1±1.5mmHg, ∆HR=-2.1±1.6 bpm, n=3 rats, 7injections). None of the doses produced a significant change in MAP or HR compared with the saline group and pre-injection values. A sample of the arterial pressure and HR tracings before and after the injection of 1000 pmol/100nl baclophen are shown in figure 4 and the maximum MAP and HR changes are shown in figure 2.

*Cardiovascular Responses to Phaclofen Microinjected into the VTA*

**Figure 5:**
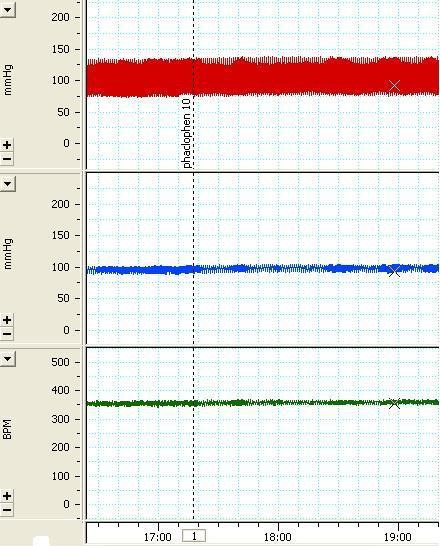
The tracing of the arterial pressure (mmHg) and heart rate (beat per minute , bpm)depicting the effects of unilateral microinjection of phaclofen 1000 pmol/100 nl the arrow indicates the injection time.

Two different doses (500 and 1000 pmole/50 nl) of phaclofen, a GABA_B_ receptor antagonist, were injected into the VTA (basal MAP=86.3±3.9 and basal HR=363.7±5.9). Tests carried out included; 500 pmol/100 nl (∆MAP=-0.68±1.25 mmHg, ∆HR=-0.56±1.03 bpm, n=10 rats, 21 injections), 1000 pmol/100 nl (∆MAP=1.5±1.2 mmHg, ∆HR=0.21±1.5 bpm, n=7 rats, 19 injections). None of the applied doses caused a significant change in MAP or HR compared with the phaclofen solvent group and pre-injection values. A sample of the arterial pressure and HR tracings before and after the injection 1000pmol/100nl phaclofen is shown in figure 5 and the maximum MAP and HR changes are shown in figure 2.

**Figure 6:**
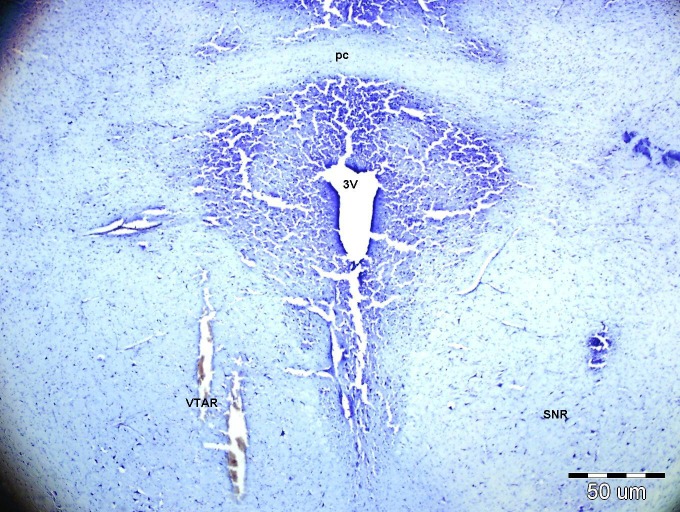
Photomicrograph of coronal section of the brain at the VTA level (Bregma: 4.8 mm caudal to bregma). The injection site was placed in the rostral portion of VTA. 3V: 3rd ventricle, pc: posterior commissure, SNR: substantia nigra, reticular part, VTAR: ventral tegmental area, rostral part. Calibration 50 μm

**Figure 7:**
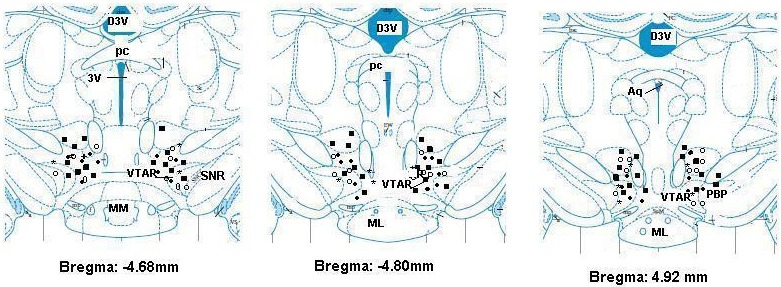
Schematic coronal sections of rat brain adopted from an atlas,22 showing the injection sites of bicuculline (filled squares), muscimol (filled circles) baclofen (open circles) and phaclofen (cross symbols). 3V: 3rd ventricle, Aq: aqueduct, D3V: dorsal 3rd ventricle: ML medial mammillary nucleus, lateral part, MM: medial mammillary nucleus, medial part, PBP: parabrachial pigmented nucleus of the VTA, pc: posterior commissure, SNR: substantia nigra, reticular part, VTAR: ventral tegmental area, rostral part.

A photomicrograph of the VTA is shown in figure 6 that shows two injection sites in the VTA. The distribution of the injection sites is shown in figure 7.

## Discussion

In the present study we demonstrated that GABAergic neurons play a major role in the cardiovascular responses of the VTA. Two regions of the VTA are reported to contain glutamic acid decarboxylase (GAD-an enzyme critical for GABA synthesis).^12^ The regions in this experiment are the same regions containing GAD enzymes. These areas correspond to the regions in our experiments (figures 6 and 7). The most responsive site was found to be in the rostral VTA 4.7-5.0 mm caudal to bregma which corresponds with GAD positive neurons.^12^

Microinjection of bicuculline, a GABA_A_ receptor antagonist, produced dose dependent pressor responses concomitant with marked bradycardia (figures 2 and 3). However, different doses of muscimol, a GABA_A_ receptor agonist, had no significant effect on blood pressure or heart rate. The pressor effect of BMI as well as insignificant response to muscimol injections implies that there should be a tonic release of GABA inhibiting the cardiovascular function via GABAA receptor. This is either by interneurons which are dispersed among the dopamine neurons^13^^,^^19^ or via GABAergic inputs to the VTA such as direct inputs from the nucleus accumbense,^19^^,^^23^ ventral pallidum,^23^^,^^24^ reticulate parts of substantia nigra^13^^,^^25^ and the bed nucleus of the stria terminalis.^26^ GABAergic neurons in some of these nuclei exert the same response as the VTA, for example the GABAergic system of the bed nucleus of the stria terminalis is tonically active and decreases the arterial pressure and heart rate.^27^ There is a GABAergic projection from VTA/SN to ventrolateral periaquductal gray region (PVGVL) and dorsal raphe nucleus (DR).^28^ Microinjection of GABA blocking agent, picrotoxin into the PVGVL/DR eliminated the cardiovascular depressor response by glutamate stimulation of VTA/SN.^28^

In an electrophysiological study that activation of GABA_A _receptor mediated post synaptic current (IPSC_s_) were recorded from dopaminergic neurons of the VTA in rats.^29^ Also the activation of presynaptic GABAA receptor facilitates further GABA release in VTA.^29^ These results provide the evidence that GABA mediated its inhibition through GABA A receptors via both dopaminergic and nondopaminergic neurons within the VTA.^30^

Microinjection of phaclofen a GABA_B_ receptor antagonist, and baclofen a GABA_B_ receptor agonist had no significant effect on AP and HR. Lack of response to phaclofen, demonstrated that the GABA cardiovascular responses are not mediated through GABA_B_ receptors. However, this result cannot rule out the presence and other possible effects of GABA_B_ receptors. For example, GABAB receptors are present on GABAergic terminals synapsing on dopaminergic neuron of the VTA; activation of these receptors presynaptically attenuates the release of GABA in the VTA.^29^ Furthermore, stimulation of GABAB receptors in the VTA blocked opiate-induced motor stimulation and motor sensitization by inhibiting the nucleus accumbens neurons.^16^ In addition, GABAB receptor of the VTA is involved in expression of morphine-induced conditioned place preference in rats.^31^

The bradycardic response to increase of BP implies that the VTA might be a part of the central baroreflex pathways. Experimental evidence has suggested that the DA neurons may be regulated by input from arterials baroreceptors.^3^^,^^32^^,^^33^ This proposition is based on the finding that denervation of arterial baroreceptors caused decrease in dopamine content and in the activity of dopamine biosynthetic enzyme tyrosine hydroxylase in the striatum part of the VTA.^34^ Some evidence suggests that dopamine neurons are under tonic inhibition of the GABAergic afferents;^20^ the GABAergic neurons of the VTA might be responding to baroreceptor input.

In summary, for the first time it was demonstrated that GABAergic neurons of the VTA are involved in cardiovascular responses through the activation of GABAA but not GABAB receptors. Theses neurons are tonically active under normal conditions. 
